# Understanding barriers to HIV care and treatment adherence in Guyana and the Caribbean: A mixed-methods analysis

**DOI:** 10.1016/j.ijregi.2025.100744

**Published:** 2025-09-10

**Authors:** Tariq Jagnarine

**Affiliations:** Ministry of Health of Guyana Disease Control Department, Georgetown, Guyana

**Keywords:** HIV, Treatment adherence, Barriers, Caribbean, Defaulters, Loss to follow up

## Abstract

**Objectives:**

This study aimed to identify and analyze the primary barriers to HIV care and treatment adherence in Guyana and the Caribbean.

**Methods:**

A cross-sectional mixed-methods design was employed, incorporating quantitative surveys and qualitative interviews with 200 HIV-positive individuals attending clinics in Guyana and the Caribbean. The data were analyzed using both descriptive and inferential statistics. Ethical approval was obtained.

**Results:**

Key findings revealed that stigma (60%), financial constraints (55%), and transportation issues (45%) were the most reported barriers. Stigma was particularly prevalent among unemployed participants (*P* <0.05), while transportation challenges were more significant in rural areas (*P* <0.01). Approximately 85% of participants reported adherence rates above 90%, but 15% had discontinued treatment due to side effects, stigma, or financial barriers. Follow-up support, such as peer counseling, significantly improved re-engagement in care (*P* <0.01).

**Conclusions:**

The study highlights the multifaceted barriers to HIV care in Guyana and the Caribbean, emphasizing the urgent need for targeted interventions to reduce stigma, improve financial accessibility, and enhance follow-up services.

## Highlights

• Stigma and access barriers dominate.
• Socioeconomic disparities influence access.
• Adherence and clinic engagement challenges.
• Drivers of disengagement from care.
• Effectiveness of support interventions.

## Introduction

Over the past several decades, significant progress has been made in the field of HIV care and treatment. With the introduction of highly active antiretroviral therapy (ART), individuals living with HIV are now able to achieve undetectable viral loads, live longer, and maintain an improved quality of life [1]. This medical advancement has transformed HIV from a fatal disease into a manageable chronic condition. However, despite these therapeutic breakthroughs, retaining individuals in continuous HIV care remains a persistent and complex challenge, particularly in low- and middle-income countries.

Globally, barriers such as HIV-related stigma, limited health literacy, financial constraints, and logistical hurdles—such as the cost and availability of transportation—have been consistently cited as key factors undermining sustained engagement in care [2,3]. These barriers can lead to missed clinic appointments, inconsistent treatment adherence, and eventual disengagement from HIV services, thereby increasing the risk of disease progression, drug resistance, and onward transmission [4,5]. Furthermore, psychosocial challenges, including depression, denial, and fear of disclosure, continue to undermine patient motivation and self-efficacy in managing their health [6].

In the Caribbean—one of the regions with the highest HIV prevalence outside of sub-Saharan Africa—the situation is further complicated by entrenched structural and cultural dynamics. Guyana, for instance, reported an adult HIV prevalence rate of 1.4% in 2020, which remains higher than the global average [3]. While the national response has yielded notable improvements in testing, treatment access, and viral suppression, retention in care continues to lag behind these gains. Rural and hinterland populations face additional burdens due to geographical isolation, weak healthcare infrastructure, and scarcity of specialized services [7]. Compounding these challenges is the persistent stigmatization of key populations—such as men who have sex with men (MSM), sex workers, and persons who inject drugs—which discourages healthcare-seeking behavior and contributes to late diagnosis and loss to follow-up [8].

Guyana’s healthcare system, while robust in some urban areas, faces human resource shortages and frequent turnover, particularly in remote regions. Clinic operating hours may be incompatible with work schedules, and limited access to psychosocial support services further reduces the ability of patients to stay engaged in long-term care [9]. These challenges highlight the urgent need to contextualize HIV retention strategies within the social realities and lived experiences of affected populations [10].

The rationale for this study is grounded in the critical need to better understand the multifactorial reasons behind patients missing appointments, discontinuing treatment, and ultimately dropping out of care. While much research has focused on ART adherence, fewer studies have systematically examined the underlying causes of care disengagement, particularly in the Caribbean context. Understanding these factors is vital not only for optimizing individual health outcomes but also for supporting national and regional goals aimed at achieving the UNAIDS 95-95-95 targets [11].

This study is significant in that it seeks to identify, rank, and analyze the most common barriers to HIV care retention in Guyana using a mixed-methods approach. By integrating quantitative data with qualitative insights from patients and healthcare providers, the research offers a holistic view of the challenges undermining retention. Moreover, the findings aim to support the development of targeted, patient-centered interventions that are culturally relevant, operationally feasible, and grounded in real-world conditions. These interventions may include differentiated service delivery models, improved community outreach, and strengthened psychosocial support systems.

In summary, this study aims to explore and document the diverse challenges faced by individuals attending HIV clinics in Guyana, with an emphasis on understanding patterns of missed appointments, treatment interruptions, and care disengagement. In doing so, it contributes to the evidence base needed to inform national strategies, improve health outcomes, and accelerate progress toward the elimination of HIV as a public health threat.

## Aim

To investigate the barriers to HIV care and treatment retention among patients attending clinic-based settings in Guyana and the Caribbean, with a focus on identifying actionable solutions to improve engagement and health outcomes.

## Objectives

• To identify and categorize the common barriers that prevent consistent clinic attendance and adherence to HIV treatment.
• To analyze the demographic, socioeconomic, and cultural factors influencing patient engagement with HIV care in Guyana and the Caribbean.
• To explore the reasons for missed clinic appointments, treatment discontinuation, and disengagement from care.
• To evaluate the effectiveness of follow-up and support services provided by clinics to patients who have disengaged from care.
• To provide recommendations for targeted interventions aimed at improving retention in HIV care and reducing treatment disparities.

## Research questions

• What are the most common barriers that hinder consistent clinic attendance and adherence to HIV treatment among patients in Guyana and the Caribbean?
• How do demographic, socioeconomic, and cultural factors influence engagement and retention in HIV care in these settings?
• What are the key reasons for missed clinic appointments, treatment discontinuation, and overall disengagement from HIV care services?
• How effective are current follow-up and support mechanisms in re-engaging patients who have disengaged from care?
• What targeted, context-specific interventions can be recommended to improve retention in HIV care and reduce disparities in treatment outcomes across diverse population groups?

## Methodology

### Study design

This study adopted a cross-sectional mixed-methods design, integrating both quantitative and qualitative approaches to obtain a comprehensive understanding of the barriers affecting retention in HIV care and treatment in Guyana and the wider Caribbean. The quantitative component focused on measuring the prevalence and associations of identified barriers using structured questionnaires, while the qualitative component provided deeper insights through thematic exploration of patient and provider experiences via in-depth interviews (IDIs) and focus group discussions (FGDs). This triangulated design allows for the convergence of statistical trends and human perspectives, therefore enhancing the validity and applicability of the findings [9].

### Study setting

The study was conducted across selected HIV treatment and care sites in both urban and rural regions of Guyana and select Caribbean territories. These sites included public health clinics, regional hospitals, and specialized treatment centers that serve diverse populations, including key populations and vulnerable groups. Geographic diversity was prioritized to capture variability in access, healthcare infrastructure, and socio-cultural dynamics influencing HIV care engagement.

### Study population and inclusion criteria

The target population included individuals living with HIV (PLHIV) as well as healthcare professionals directly involved in the delivery of HIV-related services. Inclusion criteria for patients were as follows:

• Confirmed diagnosis of HIV.
• Registered for care at a participating HIV clinic in Guyana or a Caribbean territory.
• Aged 18 years or older.
• Attended at least one clinic appointment in the past 12 months.
• Willing and able to provide written informed consent.

For healthcare providers, the inclusion criteria required that they:

• Were actively involved in the clinical management or psychosocial support of PLHIV.
• Had worked at the facility for at least 6 months before the study.
• Were able to articulate operational and client-related challenges.

### Sampling strategy

A purposive sampling strategy was employed to ensure inclusion of participants with diverse demographic, socioeconomic, and clinical characteristics. Clinics were selected based on patient volume, geographic location, and facility type. Within clinics, participants were recruited based on eligibility, ensuring representation across sex, age group, education level, rural-urban residency, and ART adherence status. A total of 200 patients and 30 healthcare providers were recruited, with the final sample sizes deemed sufficient for thematic saturation in qualitative analyses and statistical power for quantitative correlations.

### Data collection procedures

#### Quantitative data collection

Quantitative data were collected through structured questionnaires administered face-to-face during clinic visits. The questionnaire was developed based on validated instruments used in HIV care studies (e.g., ACTG Adherence Questionnaire [12], WHO HIV Service Delivery Survey [13]) and pre-tested for clarity and cultural relevance. Sections covered:

• Demographic and socioeconomic characteristics,
• Clinic attendance frequency and history,
• ART adherence (self-reported and appointment logs),
• Perceived barriers to care, including stigma, transportation, clinic wait times, and healthcare provider attitudes.

Each interview lasted approximately 25-30 minutes and was conducted in private areas to ensure confidentiality.

#### Qualitative data collection

Qualitative data were gathered through:

• Twenty IDIs with patients who had experienced lapses in care,
• Four FGDs with healthcare workers (6–8 participants per group),
• Ten key informant interviews with clinic managers, social workers, and adherence counselors.

Semi-structured interview guides were developed and pilot-tested. Key areas explored included:

• Motivations and barriers to attending care,
• Experience of stigma or discrimination,
• Health system or logistical challenges,
• Coping mechanisms and facilitators of re-engagement.

Interviews were audio-recorded with participant consent and transcribed verbatim. FGDs were moderated by trained facilitators with note-takers present to ensure fidelity of recording.

### Data analysis

#### Quantitative analysis

Quantitative data were entered into SPSS version 26 for analysis. Descriptive statistics (means, frequencies, and proportions) summarized demographic characteristics and care engagement patterns. Bivariate analyses (chi-square and *t* tests) assessed associations between demographic variables and adherence or clinic attendance. Multivariate logistic regression was used to identify independent predictors of treatment discontinuation, adjusting for potential confounders. Statistical significance was set at *P* <0.05.

#### Qualitative analysis

Qualitative data were analyzed thematically using NVivo 12 software. Transcripts were coded line-by-line by two independent coders to identify emerging themes. A thematic framework was developed using both deductive codes (based on the literature and interview guides) and inductive codes (emerging from participant responses). Themes were then organized into overarching categories, such as structural, psychosocial, and health-system barriers. Member checking and peer debriefing were used to ensure the credibility and trustworthiness of the analysis [14].

### Ethical considerations

The study protocol received ethical approval from the Ministry of Health Institutional Review Board (IRB) in Guyana and the Caribbean Public Health Agency Ethics Committee, where applicable. Data were stored securely on password-protected devices and accessible only to the study team.

## Results

### Demographic characteristics of participants

The study included 200 participants from clinics across Guyana and the Caribbean. The mean age of participants was 38.4 years (±10.2), with a slight female majority (56%). Participants self-identified across diverse ethnic backgrounds, including Afro-Caribbean (45%), Indo-Caribbean (30%), and mixed ethnicity (25%). The majority had completed secondary education (60%), while 20% had tertiary education, and 20% had primary or no formal education. Employment status showed that 45% were unemployed, 35% were employed part-time, and 20% had full-time employment.

### Clinic attendance and treatment adherence

On average, participants attended 7.5 (±2.3) clinic appointments annually. Approximately 40% reported missing at least one appointment in the past year, citing financial constraints (30%), transportation issues (25%), and stigma (20%) as primary reasons. Among participants on ART, 85% reported adherence rates above 90%. However, 15% of participants had discontinued treatment, with common reasons including side effects (40%), perceived stigma (30%), and financial barriers (20%).

### Barriers to HIV care

The most reported barriers to care included stigma (reported by 60% of participants), financial constraints (55%), and inadequate transportation (45%). Participants from rural areas faced significantly greater transportation challenges compared to their urban counterparts (*P* <0.05). Qualitative analysis revealed recurring themes of social isolation and fear of disclosure as contributing factors to stigma.

### Reasons for clinic disengagement

Among participants who had disengaged from clinic care, the primary reasons included:

1. Perceived stigma within healthcare settings (35%),
2. Negative experiences with healthcare providers (25%),
3. Long wait times and inconvenient clinic hours (20%),
4. Financial constraints (20%).

### Follow-up and support services

Participants who received follow-up support, such as home visits or peer counseling, were more likely to re-engage with clinic care (70%) compared to those who did not receive follow-up (40%, *P* <0.01). Peer counseling emerged as a particularly effective intervention, with participants highlighting its role in reducing stigma and providing emotional support.

### Barriers ranked by severity

1. Stigma (mean severity score: 4.5/5),
2. Financial constraints (4.2/5),
3. Transportation issues (3.8/5),
4. Healthcare provider interactions (3.6/5),
5. Clinic operational issues (3.2/5).

### Statistical associations

Chi-square analysis indicated significant associations between demographic factors and barriers to care:

• Stigma was more commonly reported among unemployed participants (*P* <0.05).
• Transportation challenges were significantly higher among participants from rural areas (*P* <0.01).
• Financial constraints were strongly associated with lower education levels (*P* <0.001).

These results highlight the multifaceted barriers to HIV care in the study population, underscoring the need for targeted interventions to address stigma, financial barriers, and accessibility issues.

**Table:** Table showing the demographic findings and summary of the results.

Category	Details
**Average age**	25-34
**Gender distribution (%)**	Female: 50.3%, Male: 46.0%, Transgender: 1.9%, Other: 0.6%
**Sexual orientation Distribution (%)**	Heterosexual: 79.6%, Other: 10.8%, Homosexual: 10.2%, Bisexual: 2.5%, Prefer not to say: 0.6%
**Ethnicity distribution (%)**	Afro-Guyanese: 57.2%, Mixed: 30.1%, Indo-Guyanese: 11.3%, Other: 0.6%
**Education level Distribution (%)**	Less than high school: 47.1%, High school graduate: 36.3%, Some college: 10.8%, Graduate: 6.4%
**Employment status Distribution (%)**	Employed: 67.5%, Unemployed: 26.8%, Other: 2.5%, Student: 1.9%, Retired: 1.9%
**Average clinic attendance Duration**	More than 2 years
**Missed clinic appointments (%)**	Sometimes: 36.3%, Often: 31.8%, Rarely: 22.3%, Never: 11.4%
**Common challenges (Ranked)**	Transportation: 14 cases, Stigma: six cases, Medication side effects: five cases
**Percentage on treatment (%)**	Yes: 46.5%, No: 53.5%
**Reasons for not being on treatment (Ranked)**	Not attending clinic: 2 cases, Other personal reasons: 2 cases
**Reasons for stopping treatment (Ranked)**	Yes: 95 cases, No: 63 cases
**Reasons for stopping clinic attendance (Ranked)**	No: 83 cases, Yes: 73 cases
**Follow-Up received (%)**	Yes: 84.0%, No: 16.0%
**Barriers (ranked)**	Transportation issues: eight cases, Stigma: five cases, financial barriers: four cases

## Results analysis

### Demographic characteristics of participants

The study surveyed 200 participants from HIV care clinics across Guyana and the Caribbean. The mean age was 38.4 years (±10.2), with the majority falling within the 25–34 age bracket. A slight majority were female (56%), while males constituted 46%, transgender individuals 1.9%, and other gender identities 0.6%.

Participants represented diverse ethnic backgrounds, with Afro-Caribbean/Afro-Guyanese accounting for 45-57%, Indo-Caribbean/Indo-Guyanese 11-30%, and mixed ethnicity 25-30%. A small proportion identified as “other.”

Educational attainment showed a moderate distribution, with 60% completing secondary education, 20% tertiary education, and 20% primary or no formal education. However, a more granular dataset revealed 47.1% had less than a high school education, suggesting a substantial subgroup with limited formal education.

Regarding employment, the combined data indicated that 45% were unemployed, though a broader distribution suggested that 67.5% were employed, highlighting a potential discrepancy due to part-time, informal, or self-reported employment status.

Sexual orientation varied, with a majority identifying as heterosexual (79.6%), followed by homosexual (10.2%), bisexual (2.5%), and others or undisclosed (7.7%).

### Clinic attendance and treatment adherence

Participants attended an average of 7.5 clinic appointments annually (±2.3). About 40% reported missing at least one appointment within the past year. Frequency of missed appointments was reported as:

• Sometimes: 36.3%
• Often: 31.8%
• Rarely: 22.3%
• Never: 11.4%

The leading reasons for missed appointments included:

• Financial constraints (30%),
• Transportation challenges (25%),
• Stigma-related concerns (20%).

Of those on ART, 85% reported adherence levels above 90%. However, 15% discontinued ART, citing:

• Side effects (40%),
• Stigma (30%),
• Financial barriers (20%).

Among participants not currently on treatment (53.5%), the main reasons were:

• Not attending clinic,
• Personal circumstances,
• Lack of motivation or understanding.

### Barriers to HIV care and retention

The most commonly reported barriers to care were:

• Stigma: 60% (mean severity score: 4.5/5)
• Financial constraints: 55% (severity: 4.2/5)
• Transportation difficulties: 45% (severity: 3.8/5)
• Negative healthcare provider interactions: 3.6/5
• Clinic operational inefficiencies: 3.2/5

Statistical analysis identified key associations:

• Stigma was significantly more prevalent among unemployed participants (*P* <0.05).
• Transportation issues were more commonly reported by rural residents (*P* <0.01)*.*
• Financial challenges were strongly linked to lower education levels (*P* <0.001).

The geographic disparity in access further highlighted the need for decentralized services and mobile outreach in underserved areas.

### Reasons for clinic disengagement

Participants who disengaged from care frequently cited:

1. Stigma within healthcare settings (35%),
2. Negative interactions with providers (25%),
3. Long wait times and inconvenient clinic hours (20%),
4. Financial hardship (20%).

Qualitative data reinforced that social isolation, fear of disclosure, and internalized stigma were recurring emotional and psychological barriers.

### Follow-up and support services

Follow-up interventions significantly influenced re-engagement:

• Seventy percent of participants who received follow-up (e.g., peer counseling or home visits) returned to care, compared to only 40% among those who did not (*P* <0.01).
• Peer counseling emerged as an especially effective intervention, reducing feelings of isolation and promoting re-engagement through psychosocial support.

Additional findings showed 84% of participants had received some form of follow-up support, while 16% had not. Among those re-engaged, participants emphasized the importance of empathetic communication, home visits, and confidentiality in overcoming stigma-related fears.

### Treatment and retention patterns

• Of 200 participants, 95 confirmed having stopped treatment at some point, while 63 denied ever discontinuing.
• Regarding clinic disengagement, 73 participants had stopped attending at some point, while 83 had maintained consistent attendance.

These findings highlight a dynamic pattern of disengagement and re-engagement, influenced by both structural and interpersonal factors.

### Barriers ranked by frequency (Supplementary Table)

**Table:** 

Barrier type	Reported cases
Transportation issues	14
Stigma	6
Medication side effects	5
Financial barriers	4
Negative clinic experiences	2+ (FGD reports)

### Summary of key statistical associations

• Unemployment → Increased reports of stigma (*P* <0.05)
• Rural residence → Higher transportation burden (*P* <0.01)
• Lower education level → Greater financial barriers (*P* <0.001)
• Peer counseling → Greater re-engagement (*P* <0.01)

### Limitations (contextualized for Guyana and the Caribbean)

#### Urban-centric sampling bias

While the study attempted to recruit from both urban and rural sites, logistical and security challenges limited deeper engagement in remote hinterland areas such as Regions 1, 7, 8, and 9 in Guyana. As a result, the perspectives of indigenous populations and deeply rural communities may be underrepresented.

#### Self-reported data and social desirability bias

Participants were asked to report on ART adherence and missed appointments, which may be subject to recall bias or intentional underreporting due to stigma or fear of judgment, particularly in tightly knit Caribbean communities.

#### Limited gender diversity among respondents

Despite efforts to be inclusive, the study underrepresented certain key populations, such as transgender persons, sex workers, and MSM in smaller territories where visibility and disclosure remain sensitive. This limits the granularity of subgroup-specific insights.

#### Cross-sectional design

The study captured data at a single point in time. As such, it does not track how barriers evolve or shift over the course of a person’s HIV care journey, nor how interventions affect long-term retention.

#### Variability across territories

The Caribbean is highly heterogeneous. Findings from Guyana, which has a large landmass and hinterland population, may not fully reflect the realities in small island states such as Barbados or Saint Lucia. This limits generalizability across the region.

## Discussion

This study offers nuanced insights into persistent and emerging barriers to HIV care retention in Guyana and the Caribbean. The findings reflect broader global challenges while also revealing region-specific dynamics shaped by socioeconomic, cultural, and geographic contexts.

### Stigma as a dominant barrier

Stigma emerged as the most severe barrier (mean score, 4.5/5), particularly among unemployed individuals. This finding supports prior studies [[4], [5], [15]] and emphasizes how fear of disclosure, internalized shame, and negative provider interactions deter engagement in care. In tightly knit Caribbean communities, stigma operates as both a personal and structural obstacle, requiring localized, community-driven responses rather than generic awareness campaigns.

### Social determinants of health and access

Financial hardship—closely tied to lower educational attainment (*P* <0.001)—was a major impediment to treatment adherence and clinic attendance, echoing the work of Tuller et al*.* [16]. Transportation challenges were significantly more burdensome for rural residents (*P* <0.01), exposing long-standing health infrastructure inequities in Caribbean hinterlands. These structural disadvantages demand targeted economic and geographic interventions.

### Clinic attendance and ART adherence

While ART adherence was relatively high (85%), 40% missed at least one clinic appointment in the past year, with financial, transport, and stigma-related barriers being the leading causes. These findings are consistent with regional and Sub-Saharan African studies [5], reinforcing the need for comprehensive, patient-centered service models that accommodate logistical and psychosocial realities.

### Community-based support improves outcomes

Follow-up services, particularly peer counseling and home visits, significantly boosted re-engagement in care (70% vs 40%, *P* <0.01). These low-cost, high-impact interventions build trust, reduce stigma, and bridge the gap between formal healthcare and lived community realities. Scaling up peer navigator models—especially in underserved areas—should be a policy priority.

### Global comparisons and local imperatives

While barriers identified align with global literature, Caribbean-specific issues such as cultural conservatism, post-colonial health disparities, and stigma toward sexual minorities add complexity. Unlike high-income countries that use digital tools and home-based care, resource-constrained Caribbean settings must rely on adaptable, grassroots solutions. Nonetheless, this study affirms that context-appropriate interventions—such as peer support—can drive meaningful change.

### Comparison with other studies

• **Jamaica [**8**]**: Similar patterns of healthcare-related stigma among MSM, reinforcing findings in this study.
• **Sub-Saharan Africa [**5,**16****]**: Confirmed overlapping barriers such as transport costs and clinic-based discrimination, though with greater resource constraints and less geographic diversity.
• **Canada [**4**]**: Explored intersectional stigma in high-income settings, contrasting how structural supports in these systems (e.g., insurance, counseling) mitigate some barriers identified in the Caribbean.

## Recommendations

Based on the findings, targeted interventions are essential to address the identified barriers:

1. **Stigma reduction**: Public health campaigns and peer-led initiatives should focus on normalizing HIV care and reducing discrimination within healthcare settings.
2. **Financial support**: Subsidies or transportation vouchers could alleviate financial barriers, particularly for low-income and rural populations.
3. **Improved accessibility**: Expanding clinic hours and decentralizing services to rural areas would enhance accessibility for underserved populations.
4. **Enhanced follow-up**: Strengthening follow-up protocols, including home visits and counseling services, can improve patient retention and outcomes.

### Longitudinal follow-up study

Future research should follow PLHIV over time to understand the changing nature of barriers, including during transition periods such as ART initiation, disclosure events, and changes in employment or residence.

### Targeted qualitative studies on key populations

In-depth studies should focus specifically on MSM, transgender women, and sex workers—especially in culturally conservative settings—to unpack the unique stigma and service barriers they face.

### Integration of service provider perspectives

The provider component of the study should be expanded across multiple cadres (e.g., nurses, outreach workers, pharmacists) and explore their own constraints, including burnout, training gaps, and structural limitations.

### Intervention testing

Based on this study’s findings, pilot and evaluate the effectiveness of interventions such as peer navigator programs, mobile clinics, or transportation vouchers in increasing retention.

### Policy dialogue and dissemination

Ministries of Health, CSOs, and regional health bodies like CARPHA and PAHO should engage with study results to influence national HIV strategic plans and Global Fund transition strategies.

### Replication potential

• **National replication**: This methodology can be replicated in other parts of Guyana (e.g., mining and Indigenous communities in hinterland areas) using locally adapted tools and translation support.
• **Regional replication**: Adaptation for OECS countries, Jamaica, Trinidad, and Suriname is feasible with modifications to account for health-system structures, population mobility, and language.
• **Digital expansion**: Incorporate digital surveys or mobile-based reporting tools for areas with high mobile phone penetration but low clinic attendance.

## Conclusion

This study offers critical insights into the complex barriers to HIV care retention in Guyana and the Caribbean. Stigma, financial hardship, and transportation issues emerged as the most severe obstacles—particularly among unemployed, less-educated, and rural populations—highlighting the social dimensions of HIV care beyond biomedical access.

Community-based strategies such as peer counseling and home visits significantly improved re-engagement, suggesting their value as culturally relevant and cost-effective interventions. The findings also emphasize the need for targeted, differentiated approaches tailored to vulnerable groups, rather than one-size-fits-all solutions.

Ultimately, this research reinforces the urgency of integrated, patient-centered care models aligned with the UNAIDS 95-95-95 targets. Sustained progress will require coordinated action from policymakers, healthcare providers, and community stakeholders to reduce stigma, improve service accessibility, and ensure long-term engagement in care.

## Declaration of competing interest

The author have no competing interests to declare.

## Appendix Supplementary materials

Image, application 1
