# Supplementary material for: Understanding barriers to HIV care and treatment adherence in Guyana and the Caribbean: A mixed-methods analysis
Source: IJID Reg. 2025 Sep 10;17:100744. doi: 10.1016/j.ijregi.2025.100744 (PMC12537562; doi:10.1016/j.ijregi.2025.100744)
Supplement: Supplementary file 1 [file mmc1.docx]

#### **Barriers Ranked by Frequency (Supplementary Table)**

| **Barrier Type** | **Reported Cases** |
| --- | --- |
| Transportation Issues | 14 |
| Stigma | 6 |
| Medication Side Effects | 5 |
| Financial Barriers | 4 |
| Negative Clinic Experiences | 2+ (FGD reports) |
